# The Mental Health Burden of the COVID-19 Pandemic on Physical Therapists

**DOI:** 10.3390/ijerph17103723

**Published:** 2020-05-25

**Authors:** Seoyon Yang, Sang Gyu Kwak, Eun Jae Ko, Min Cheol Chang

**Affiliations:** 1 Department of Rehabilitation Medicine, Ewha Woman’s University Seoul Hospital, Seoul 07804, Korea; 00993s@eumc.ac.kr; 2 School of Medicine, Ewha Woman’s University, Seoul 07804, Korea; 3 Department of Medical Statistics, College of Medicine, Catholic University of Daegu, Daegu 42472, Korea; sgkwak@cu.ac.kr; 4 Department of Rehabilitation Medicine, Asan Medical Center, University of Ulsan College of Medicine, Seoul 05505, Korea; gonjae0610@gmail.com; 5 Department of Rehabilitation Medicine, College of Medicine, Yeungnam University, Daegu 42415, Korea

**Keywords:** COVID-19, coronavirus, physical therapists, anxiety, depression, mental health

## Abstract

We evaluated the mental health burden of coronavirus disease (COVID-19) on physical therapists, including their stress and anxiety levels, who were at risk of developing psychological distress and other mental health symptoms. A questionnaire survey was conducted with physical therapists of three university hospitals in South Korea on 10 April 2020. The questionnaires evaluated the presence of anxiety and depression in the respondents. Among the 65 physical therapists who completed our survey, 21 (32.3%) and 12 (18.5%) physical therapists reported having symptoms of anxiety and depression, respectively. If a physical therapist was living with a ≤6-year-old infant or child, the possibility of the presence of anxiety was significantly higher. The risk of depression among those who were in their 30 s and 50 s was significantly higher than among those in their 20 s. Thus, physical therapists living with a ≤6-year-old infant or child and those in their 30 s and 50 s need special attention.

## 1. Introduction

Coronavirus disease (COVID-19) is an infectious disease caused by severe acute respiratory syndrome coronavirus 2 (SARS-CoV-2). It was first identified in December 2019 in Wuhan, China, and has been spreading domestically and globally ever since. In South Korea, after the identification of the first patient on 20 January 2020, the number of confirmed cases has been constantly increasing. The South Korean government and the Korea Centers for Disease Control and Prevention (K-CDC) reported that as of 13 April 2020, the total number of confirmed cases of COVID-19 had increased to 10,512. This critical, infectious public health concern has disrupted the usual routine of life [1], caused panic and fear due to strict isolation measures, and caused uncertainty and stigmatization [2]. People feel unsafe and anxious when facing outbreaks of infectious disease when the cause, progression, and outcomes of the disease are unclear [3]. The continuous spread of the epidemic has significant potential for psychological contagion and can result in widespread fear, anxiety, and other psychological problems [4].

Healthcare providers, who are on the front line of fighting this devastating situation, are under not only the risk of death from infection but also insurmountable psychological pressure. People who work in hospitals are more exposed to infectious diseases than the general public. After the 2003 SARS outbreak, healthcare workers reported worries about the contagion and infection of their families and friends [5], as well as high levels of anxiety, stress, and depression [6]. Similar concerns about the psychological impact of COVID-19 on healthcare providers are arising [7]. A recent study reported that medical health workers suffered from more psychosocial problems, including insomnia, anxiety, and depression, than nonmedical health workers during the COVID-19 outbreak in China [8].

Among the healthcare providers, it appears that there is likely to be substantial increases in stress and anxiety among physical therapists in the context of the COVID-19 pandemic. Person-to-person transmission of the COVID-19 infection has been widely documented [9], which occurs primarily via direct contact with infected patients or through droplets spread by infected patients coughing or sneezing [10]. Thus, it can be assumed that physical therapists are at risk of being in contact with COVID-19 patients in a rehabilitation setting. During rehabilitation programs, physical therapists have direct contact with patients, which increases the potential to be exposed to COVID-19. Many physical therapists experience work-related musculoskeletal disorders and workplace stress due to excessive amounts of work, even in the usual rehabilitation setting [11]. The likelihood of developing mental health problems may be higher during this epidemic. However, to the best of our knowledge, no detailed study on the mental health status of physical therapists has been conducted to date. This study aimed to investigate the mental health burden of COVID-19 on physical therapists, including their stress and anxiety levels.

## 2. Materials and Methods

### 2.1. Study Design and Participants

We conducted a questionnaire survey with physical therapists of three university hospitals in South Korea on 10 April 2020. We recruited all the physical therapists working in each university hospital. The exclusion criteria were previous diagnoses of psychiatric disorders and a history of confirmed infection of COVID-19; based on these criteria, no physical therapist was excluded from our study. This study was approved by the Institutional Review Board of Yeungnam university hospital (2020-05-078) and all participants provided written informed consent to be included in the study.

### 2.2. Survey Questionnaire

The questionnaire comprised three parts: epidemiology (age range, sex, family members living together, previous medical history, isolation experience due to contact with COVID-19 patients, and the presence of family members infected with COVID-19), anxiety, and depression. We checked the presence of anxiety and depression, which were measured using the Generalized Anxiety Disorder scale (GAD-7) [11] and the Patient Health Questionnaire-9 (PHQ-9) [12], respectively. GAD-7 is a seven-item anxiety scale with total scores ranging from 0 to 21 (cut-off value: ≥5), and PHQ-9 is a nine-item depression scale with total scores ranging from 0 to 27 (cut-off value: ≥10). The alpha reliabilities of GAD-7 and PHQ-9 were reported to be 0.80 and 0.85, respectively [13,14]. In primary and secondary mental health care services, GAD-7 and PHQ-9 are commonly used measures of anxiety and depression symptoms, respectively.

### 2.3. Statistical Analysis

We analyzed the presence of anxiety and depression relative to the demographic data. Chi-square and *t*-tests were conducted to evaluate the association of demographic factors with the risk of anxiety and depression. In the case of a significant association being found, binary logistic regression analysis was conducted for clearer results regarding risk factors. The acceptable statistical significance was set at *p* < 0.05.

## 3. Results

Of the 73 physical therapists working in three university hospitals (7, 33, and 33), 65 (7, 28, and 30 in each hospital) completed our survey. Twenty-one physical therapists (32.3%) had total scores of ≥ 5 on GAD-7, indicating the presence of anxiety (Table 1). The mean GAD-7 score of the 65 physical therapists was 3.6 ± 4.2. Regarding PHQ-9, 12 physical therapists (18.5%) had total scores of ≥10, indicating the presence of depression (Table 2). The mean PHQ-9 score of the 65 physical therapists was 5.0 ± 4.2.

The results revealed that physical therapists who lived with a ≤6-year-old infant or child or a ≥65-year-old person had a significantly higher risk of anxiety (*p* = 0.014) (Table 1). However, no other factors showed a statistically significant difference between physical therapists with anxiety and those without anxiety (*p* > 0.05). As shown by the binary logistic regression analysis in Table 3, if a physical therapist had a ≤6-year-old infant or child, the risk of anxiety significantly increased, reaching 6.727 times higher than for those who had neither a ≤6-year-old infant or child nor were living with a ≥65-year-old person (*p* = 0.007).

The risk of depression differed depending on the age range (*p* = 0.014) (Table 2). No other factors were statistically significant regarding the risk of depression. None of the physical therapists in their 20 s had depression. However, 7 out of 20 (35%) physical therapists in their 30 s had depression, as did 2 out of 16 (12.5%) physical therapists in their 40 s and 3 out of 8 (37.5%) in their 50 s. As shown by the binary logistic regression analysis, physical therapists in their 30 s and 50 s had a significantly higher risk of depression than those in their 20 s (Table 4). The risk of depression in physical therapists who were in their 30 s and 50 s was 22.615 and 25.2 times higher, respectively, than for those who were in their 20 s.

## 4. Discussion

Through a cross-sectional survey, this study aimed to investigate the psychological status and mental health burden among physical therapists during the COVID-19 outbreak. Overall, 32.3% and 18.5% of physical therapists reported having symptoms of anxiety and depression, respectively. Considering the National Mental Health Survey conducted on the general population by the South Korean government in 2016 showed that the average prevalence of anxiety and depression were 9.3% and 5.0%, respectively, the physical therapists who participated in our study appeared to have high rates of anxiety and depression [15]. The COVID-19 infection is likely to spread via person-to-person transmission in the community, mostly among adults [10]. Person-to-person transmission occurs through contact or droplet transmission. This may jeopardize the health of the healthcare workers, including physical therapists, who are at risk of exposure due to the nature of their occupation. Physical therapists may feel that they are vulnerable to COVID-19 infection when performing rehabilitation programs, which frequently involve direct contact with patients.

When facing the global spread of COVID-19, people are mostly concerned about their wellbeing and the health of their family members [16]. After confirmation of a COVID-19 infection, critical patients get transferred to negative pressure isolation rooms, which are designated by the government for proper treatment. The general public likely fear that they would be isolated from their families and friends, resulting in them feeling unsafe and uncertain about their wellness. In particular, our study revealed that physical therapists who were living with a ≤6-year-old infant or child were more likely to experience anxiety. In fact, physical therapists who had a ≤6-year-old infant or child had a 6.727 times higher risk of experiencing anxiety than those who did not. COVID-19 appears to be relatively rare in children, and children seem to present less severe symptoms compared to adults [17]. However, the COVID-19 infection can occur in children via intrafamilial transmission if the virus spreads within the family through infected adults [18]. The first pediatric case of COVID-19 occurred in Korea on 18 February 2020, involving a 10-year-old girl who had close contact with her uncle and mother, who were both diagnosed with COVID-19 [19]. Most of the COVID-19 infection cases in children were found among family clusters as a result of close contact with other COVID-19 cases. While in self-quarantine, infants and young children must be taken care of by their caregivers; self-isolation of young children by themselves is almost impossible [19]. Although most of the infected children had mild or difficult-to-recognize symptoms, at present, there are no specific antiviral drugs or vaccines against the COVID-19 infection [20]. Understandably, families with young children are anxious and concerned about the transmission of COVID-19.

The results of the current study also revealed that 18.5% of physical therapists had depression, occurring in 35%, 12.5%, and 37.5% of those in their 30 s, 40 s, and 50 s, respectively. None of the physical therapists in their 20 s had depression. Physical therapists in their 30 s and 50 s had a significantly higher risk of depression compared to those in their 20 s. The risk of depression in physical therapists who were in their 30 s and 50 s was 22.615 and 25.2 times higher than for those in their 20 s, respectively. Interestingly, according to age range, there was no linear change in the risk of depression for those who were in their 40 s. This correlated with the finding of a previous study, which found that the 40 s are a bridge between the lowest levels of depression and a gradual increase in depression [21].

This study has several limitations. First, the study did not employ a control group nor a longitudinal follow-up. Considering the increasing number of confirmed cases of COVID-19, the mental health burden of physical therapists could become more severe. Thus, it would be ideal to conduct a prospective study on the same physical therapists after a period, including long-term courses of anxiety and depression. Second, this study was unable to distinguish pre-existing mental health symptoms from new symptoms. Third, the study design did not include cause analysis for psychological strains, such as work-related stress. Future research is needed to address the above limitations, including the comparison of the presence of anxiety and depression between physical therapists and a control group.

## 5. Conclusions

The outbreak of COVID-19 has caused a serious emerging challenge for the general public and health professionals worldwide. If traumatized, physical therapists may continue to suffer from psychiatric symptoms, even after the outbreak [22]. Finding out the stressors related to COVID-19 and detecting early warning signs of anxiety and depression can facilitate an understanding of what is required to restore physical and mental health [23]. Thus, considering that a significant percentage of physical therapists present symptoms of anxiety and depression, the mental health of physical therapists should be constantly and cautiously monitored. It is also necessary to make an effort to protect physical therapists at risk of exposure to mitigate the fear of the COVID-19 infection. Additionally, special attention needs to be given to physical therapists living with a ≤6-year-old infant or child, as well as those in their 30 s and 50 s.

## Figures and Tables

**Table 1:** Association of demographic data of physical therapists with COVID-19 and the presence of anxiety.

Variable	Total	Anxiety	No Anxiety	*p*-Value
Total, *n* (%)	65	21 (32.3)	44 (67.7)	
Age range, *n*				0.180
20 s	21	3	18	
30 s	20	9	11	
40 s	16	6	10	
50 s	8	3	5	
Sex, *n*				0.590
Male	34	12	22	
Female	31	9	22	
Family members living together				0.014 *
None	48	11	37	
≤6-year-old infant or child	12	8	4	
≥65-year-old person	5	2	3	
Previous medical history				0.435
Yes	4	2	2	
No	61	19	42	
Isolation experience				0.587
Yes	2	1	1	
No	63	20	43	
The presence of family members confirmed with COVID-19				0.747
Yes	4	1	3	
No	61	20	41	

The *p*-values were calculated using independent *t*-tests or chi-square tests, as appropriate. * Significant difference noted in the comparison between two groups (*p* < 0.05). Values: mean ± standard deviation. Abbreviation: COVID-19—coronavirus disease. Previous medical history: hypertension—three patients, thyroid cancer—one patient.

**Table 2:** Association of demographic data of physical therapists with COVID-19 and the presence of depression.

Variable	Total	Depression	No Depression	*p*-Value
Total, *n* (%)	65	12 (18.5)	53 (81.5)	
Age range, *n*				0.014 *
20 s	21	0	21	
30 s	20	7	13	
40 s	16	2	14	
50 s	8	3	5	
Sex, *n*				0.859
Male	34	6	28	
Female	31	6	25	
Family members living together				0.066
None	48	6	42	
≤6-year-old infant or child	12	5	7	
≥65-year-old person	5	1	4	
Previous medical history				0.093
Yes	4	2	2	
No	61	10	51	
Isolation experience				0.243
Yes	2	1	1	
No	63	11	52	
The presence of family members confirmed with COVID-19				0.728
Yes	4	1	3	
No	61	11	50	

The *p*-values were calculated using independent *t*-tests or chi-square tests, as appropriate. * Significant difference noted in the comparison between two groups (*p* < 0.05). Values: mean ± standard deviation. Abbreviation: COVID-19—coronavirus disease. Previous medical history: hypertension—three patients, thyroid cancer—one patient.

**Table 3:** Binary logistic regression analysis for risk factor associated with anxiety.

Comparison	OR	95% CI Interval	*p*-Value
None vs. ≤6-year-old infant	6.727	1.699–26.636	0.007 *
None vs. ≥65-year-old person	2.242	0.332–15.168	0.408

* Significant difference noted in the comparison between two groups (*p* < 0.05). Abbreviation: OR—odds ratio, CI—confidence interval.

**Table 4:** Binary logistic regression analysis for risk factor associated with depression.

Comparison	OR	95% CI Interval	*p*-Value
20 s vs. 30 s	22.615	2.806–182.273	0.003 *
20 s vs. 40 s	6.000	0.637–56.522	0.117
20 s vs. 50 s	25.200	2.719–233.538	0.005 *

* Significant difference noted in the comparison between two groups (*p* < 0.05). Abbreviation: OR—odds ratio, CI—confidence interval.
